# Supplementary material for: Molecular Epidemiology of Multidrug-Resistant Uropathogenic Escherichia coli O25b Strains Associated with Complicated Urinary Tract Infection in Children
Source: Microorganisms. 2021 Nov 5;9(11):2299. doi: 10.3390/microorganisms9112299 (PMC8623209; doi:10.3390/microorganisms9112299)
Supplement: Supplementary file 1 [file microorganisms-09-02299-s001.zip › microorganisms-1414657-supplementary.pdf]

microorganisms MDPI

Supplementary Tables

Table S1. Primers for the amplification of virulence, resistance, and housekeeping genes from UPEC O25b clinical strains as well PCR conditions.

Gene	Primers	Primer sequence (5′ to 3′)	Size (bp)	PCR Conditions	References
O25b	*pabB-F*<br>*pabB-R*	TCC AGC AGG TGC TGG ATC GT<br>GCG AAA TTT TTC GCC GTA CTG T	347	1 cycle a 95 °C/5 min<br>35 cycles a 95 °C/1 min<br>65 °C/1 min, 72 °C/1.30 min<br>72 °C/5 min, 4 °C/ꝏ min	(1)
	trpA-F<br>*trpA-R*	GCT ACG AAT CTC TGT TTG CC<br>GCA ACG CGG CCT GGC GGA AG	427		
chuA	*chuA*-F<br>*chuA*-R	GAC GAA CCA ACG GTC AGG AT<br>TGC CGC CAG TAC CAA AGA CA	279	1 cycle a 95 °C/5 min<br>25 cycles a 95 °C/1 min<br>55 °C/1 min<br>72 °C/2 min<br>72 °C/5 min<br>4 °C/ꝏ min	(2, 3)
yjaA	*yjaA*-F<br>*yjaA*-R	TGA AGT GTC AGG AGA CGC TG<br>ATG GAG AAT GCG TTC CTC AAC	211		
TspE4.C2	*TspE4*.C2-F<br>*TspE4*.C2-R	GAG TAA TGT CGG GGC ATT CA<br>CGC GCC AAC AAA GTA TTA CG	152		
hlyA	*hlyA*-F<br>*hlyA*-R	CGT GGA CAC AGC TGC CAG CA<br>TGC AGC GTG GCG GGC ATC AT	789	1 cycle a 95 °C/5 min<br>25 cycles a 95 °C/1 min<br>56 °C/1 min<br>72 °C/1 min<br>72 °C/5 min<br>4 °C/∞ min	(2, 4)
iutD	*iutD*-F<br>*iutD*-R	TAC CGG ATT GTC ATA TGC AGA CCG T<br>AAT ATC TTC CTC CAG TCC GGA GAA G	602		
fimH	*fimH*-F<br>*fimH*-R	TGC AGA ACG GAT AAG CCG TGG<br>GCA GTC ACC TGC CCT CCG GTA	508		
csgA	*csgA*-F<br>*csgA*-R	GCC AGT ATT TCG CAA GGT GC<br>GGT GTA CAT ATC CCC TTG CTG G	750	1 cycle a 95 °C/5 min<br>30 cycles a 95 °C/1 min	(2, 4, 5)

Gene	Primers	Primer sequence (5′ to 3′)	Size (bp)	PCR Conditions	References
satA	*satA*-F<br>*satA*-R	GTT GTC TCT GGC TGT TGC<br>AAT GAT GTT CCT CCA GAG C	501	56 °C/30 seg<br>72 °C/1 min<br>72 °C/5 min<br>4 °C/∞ min	
fyuA	fyuA-F<br>*fyuA*-R	GCG CAT TTG CTG ATA CTG TTG<br>CAT CAG ACG ATA AGC ATG AGC A	272		
motB	*motB*-F<br>*motB*-R	GCG GAT TTT TCA CCG CAC GC<br>GGA TCT GCT GGC TCT GC	864	1 cycle a 95 °C/2 min<br>30 cycles 94 °C/30 seg<br>49 °C/1 min<br>72 °C/3 min<br>72 °C/5 min<br>4 °C/∞ min	(2, 5)
tosA	*tosA*-F<br>*tosA*-R	GCA CAG CAT AAC GGG AAA AT<br>CCA GCA TGT TAC CAC GAA TG	589		
fliC	*fliC*-F<br>*fliC*-R	CCA GTC TGC GCT GTC GAG<br>CAC GTT CAC GCC GTT GAA C	349		
*pap*GI	*pap*GI-F<br>*pap*GI-R	CAA CCT GCT CTC AAT CTT TAC TG<br>CAT GGC TGG TTG TTC CTA AAC AT	692	1 cycle a 94 °C/5 min<br>30 cycles 94 °C/1 min<br>53 °C/1 min<br>72 °C/1 min<br>72 °C/5 min<br>4 °C/∞ min	(4)
*pap*GII	*pap*GII-F<br>*pap*GII-R	GGA ATG TGG TGA TTA CTC AAA GG<br>TCC AGA GAC TGT TCA AGA AGG AC	562		
*pap*GIII	*pap*GIII-F<br>*pap*GIII-R	CAT GGC TGG TTG TTC CTA AAC AT<br>TCC AGA GAC TGT GCA GAA GGA C	421		
bcsA	*bcsA*-F<br>*bcsA*-R	GAA GAA TTC CTG ACG CTG GCT AA<br>TGA AAG CTT GGA ACG CAC TCA TC	2,767	1 cycle a 94 °C/5 min<br>30 cycles a 94 °C/1 min<br>56 °C/1 min, 72 °C/2 min<br>72 °C/5 min, 4 °C/∞ min	(6)
ecpA	*ecpA*-F<br>*ecpA*-R	TGA AAA AAA AGG TTC TGG CAA TAG C	510	1 cycle a 94 °C/5 min	(7)

Gene	Primers	Primer sequence (5′ to 3′)	Size (bp)	PCR Conditions	References
		CGC TGA TGA GGA GAA AGT GAA		30 cycles a 94 °C/1 min 56 °C/1 min, 72 °C/2 min 72 °C/5 min, 4 °C/∞ min	
*cnf*1	*cnf*1-F *cnf*1-R	GAA CTT ATT AAG GAT AGT CAT TAT TTA TAA CGC TG	543	1 ciclo a 94 °C/5 min 30 cycles 94 °C/1 min 39.5 °C/1 min, 72 °C/1 min 72 °C/5 min, 4 °C/∞ min	(4)
motA	*motA*-F *motA*-R	GCG GAT TTT TCA CCG CAC GC GGA TCT GCT GGC TCT GC	632	1 cycle 94 °C/5 min 25 cycles a 94 °C/1 min 62 °C/1 min, 72 °C/1 min, 72 °C/5 min	(5)
intl3	*intl*3-F *intl*3-R	GCC TCC GGC AGC GAC TTT CAG ACG GAT CTG CCA AAC CTG ACT	979	1 cycle a 94 °C/5 min 30 cycles a 94 °C/1 min 53 °C/1 min, 72 °C/1 min 72 °C/5 min, 4 °C/∞ min	(8)
intl2	*intl*2-F *intl*2-R	CAC GGA TAT GCG ACA AAA AGG T GAT GAC AAC GAG TGA CGA AAT G	788		
intl1	*intl*1-F *intl*1-R	CAG TGG ACA TAA GCC TGT TC CCC GAG GCA TAG ACT GTA	160		
RV-Int-1	*Hep*58-F *Hep*59-R	GTA GGG CTT ATT ATG CAC GC CGG GAT CCC GGA CGG CAT GCA	Variable product	1 cycle a 94 °C/5 min 30 cycles a 94 °C/30 seg 55 °C/1 min, 72 °C/4 min 72 °C/5 min, 4 °C/∞ min	(9)
RV-Int-2	*Hep*74-F *Hep*51-R	CGG GAT CCC GGA CGG CAT GCA CGA TTT GTA GAT GCC ATC GCA AGT ACG AG	Variable product		

Gene	Primers	Primer sequence (5′ to 3′)	Size (bp)	PCR Conditions	References
adk	adk-Ec-F 20 bp/53 bp<br>adk-Ec-R 20 bp/54 bp	5'-TCG TCG GCA GCG TCA GAT GTG TAT AAG AGA CAG ATG CGT ATC ATT CTG CTT GG-3'<br>5'-GTC TCG TGG GCT CGG AGA TGT GTA TAA GAG ACA GCC GTC AAC TTT CGC GTA TTT-3'	593	1 cycle a 95 °C/5 min<br>30 cycles a 95 °C/1 min<br>62 °C/1 min, 72 °C/1 min<br>72 °C/5 min, 4 °C/∞ min	This study
fumC	fumC-Ec-F<br>20 bp/53 bp<br>fumC-Ec-R 21 bp/55 bp	5'-TCG TCG GCA GCG TCA GAT GTG TAT AAG AGA CAG TAG GCT TGT TGT CTG AAG AG-3'<br>5'-GTC TCG TGG GCT CGG AGA TGT GTA TAA GAG ACA GGG TAT TTA GTC CAG TAC CCA C-3'	530	1 cycle a 95 °C/5 min<br>30 cycles a 95 °C/1 min<br>62 °C/1 min, 72 °C/1 min<br>72 °C/5 min, 4 °C/∞ min	This study
gyrB	gyrB-Ec-F 20 bp/53 bp<br>gyrB-Ec-R<br>20 bp/54 bp	5'-TCG TCG GCA GCG TCA GAT GTG TAT AAG AGA CAG AAG TGA TCA TGA CCG TTC TG-3'<br>5'-GTC TCG TGG GCT CGG AGA TGT GTA TAA GAG ACA GGC GGA ATG TTG TTG GTA AAG-3'	549	1 cycle a 95 °C/5 min<br>30 cycles a 95 °C/1 min<br>62 °C/1 min, 72 °C/1 min<br>72 °C/5 min, 4 °C/∞ min	This study
icd	icd-Ec-F 20 bp/53 bp<br>icd-Ec-R 20 bp/54 bp	5'-TCG TCG GCA GCG TCA GAT GTG TAT AAG AGA CAG ACC CCA GCC ATG CTG AAA TG-3'<br>5'-GTC TCG TGG GCT CGG AGA TGT GTA TAA GAG ACA GCC TTT GTG CAC CAG AGT CAC-3'	567	1 cycle a 95 °C/5 min<br>30 cycles a 95 °C/1 min<br>62 °C/1 min, 72 °C/1 min<br>72 °C/5 min, 4 °C/∞ min	This study
mdh	mdh-Ec-F 20b9/53 bp<br>mdh-Ec-R<br>20 bp/54 bp	5'-TCG TCG GCA GCG TCA GAT GTG TAT AAG AGA CAG GCG CAG ATG TCG TTC TTA TC-3'<br>5'-GTC TCG TGG GCT CGG AGA TGT GTA TAA GAG ACA GCA TTC GAC AAC GCC TTG TTC-3'	547	1 cycle a 95 °C/5 min<br>30 cycles a 95 °C/1 min<br>62 °C/1 min, 72 °C/1 min<br>72 °C/5 min, 4 °C/∞ min	This study

Gene	Primers	Primer sequence (5′ to 3′)	Size (bp)	PCR Conditions	References
purA	purA-Ec-F 20 bp/53 bp<br>purA-Ec-R 21 bp/55 bp	5'-TCG TCG GCA GCG TCA GAT GTG TAT AAG AGA CAG GAT CCT TGA TTA TCA CGT TGC-3'<br>5'-GTC TCG TGG GCT CGG AGA TGT GTA TAA GAG ACA GGT TTC ATC AAA CAG TTC GGT C-3'	543	1 cycle a 95 °C/5 min<br>30 cycles a 95 °C/1 min<br>62 °C/1 min, 72 °C/1 min<br>72 °C/5 min, 4 °C/∞ min	This study
recA	recA-Ec-F 20 bp/53 bp<br>recA-Ec-R 20 bp/54 bp	5'-TCG TCG GCA GCG TCA GAT GTG TAT AAG AGA CAG TGC GTT TAT CGA TGC TGA AC-3'<br>5'-GTC TCG TGG GCT CGG AGA TGT GTA TAA GAG ACA GGC TTT CTC GAT CAG CTT CTC-3'	591	1 cycle a 95 °C/5 min<br>30 cycles a 95 °C/1 min<br>62 °C/1 min, 72 °C/1 min<br>72 °C/5 min, 4 °C/∞ min	This study

Table S2. Allelic profiles, type sequences, and clonal complexes associated with MDR and XDR UPEC O25b clinical strains.

Strains	Allelic profiles							STs	CCs
	adk	*fumC*	*gyrB*	*icd*	*mdh*	*purA*	*recA*		
606U1-O25b, 807U1-O25b	10	11	4	8	8	8	770	10	CC10
173U5-O25b, 424U1-O25b, 804U3-O25b	28	33	25	29	7	11	24	62	Unknown
117U1-O25b, 118U1-O25b, 177U1-O25b, 179U-O25b, 249U-O25b, 310U5-O25b, 494U2-O25b, 502U1-O25b, 553U-O25b, 562U-O25b, 657U-O25b, 736U1-O25b	21	35	27	6	5	5	4	69	CC69
21U-O25b, 294U4-O25b, 511U1-O25b, 592U4-O25b	36	24	9	13	17	11	25	73	CC73
609U-O25b	6	11	4	10	7	8	651	93	CC168
513U-O25b	20	45	41	43	5	32	2	117	Unknown
143U-O25b	49	4	44	9	11	35	7	120	Unknown
11U-O25b, 268U1-4-O25b, 268U5-O25b, 319U-O25b, 44U-O25b, 57U-O25b, 63U2-O25b, 674U-O25b, 674U1-O25b, 678U-O25b, 702U1-O25b, 711U-O25b, 720U-O25b, 722U-O25b, 756U1-O25b, 816U-O25b, 851U-O25b, 876U3-O25b, 877U-O25b, 909U-O25b, 945U2-O25b, 955U-O25b	53	40	47	13	36	28	29	131	CC131
672U1-O25b	21	35	61	484	5	5	4	394	CC394
532U1-O25b, 557U1-O25b	35	37	29	25	4	5	73	405	CC405
992U-O25b	37	38	19	27	17	8	26	421	CC95
618U1-O25b, 618U2-O25b	6	19	15	16	9	8	7	443	CC205
561U1-O25b, 618U3-O25b	13	52	156	14	17	25	17	998	Unknown
870U-O25b	4	26	2	211	5	5	19	1177	CC38

adk (adenylate kinase), *fumC* (fumarate hydratase), *gyrB* (DNA gyrase), *icd* (isocitrate/isopropylmalate dehydrogenase), *mdh* (malate dehydrogenase), *purA* (adenylosuccinate

dehydrogenase), *recA* (ATP/GTP binding motif), Type Sequences (STs) and Clonal complexes (CCs).

References

1. Clermont, O.; Dhanji, H.; Upton, M.; Gibreel, T.; Fox, A.; Boyd, D.; Mulvey, M.R.; Nordmann, P.; Ruppé, E.; Sarthou, J.L.; et al. Rapid detection of the O25b-ST131 clone of *Escherichia coli* encompassing the CTX-M-15-producing strains. *J. Antimicrob. Chemother.* **2009**, *64*, 274–277. https://doi.org/10.1093/jac/dkp194.
2. Ochoa, S.A.; Cruz-Córdova, A.; Luna-Pineda, V.M.; Reyes-Grajeda, J.P.; Cázares-Domínguez, V.; Escalona, G.; Sepúlveda-González, M.E.; López-Montiel, F.; Arellano-Galindo, J.; López-Martínez, B.; et al. Multidrug- and extensively drug-resistant uropathogenic *Escherichia coli* clinical strains: Phylogenetic groups widely associated with integrons maintain high genetic diversity. *Front. Microbiol.* **2016**, *7*, 2042. https://doi.org/10.3389/fmicb.2016.02042.
3. Clermont, O.; Bonacorsi, S.; Bingen, E. Rapid and simple determination of the *Escherichia coli* phylogenetic group. *Appl. Environ. Microbiol.* **2000**, *66*, 4555–4558. https://doi.org/10.1128/aem.66.10.4555-4558.2000.
4. Tiba, M.R.; Yano, T.; Leite Dda, S. Genotypic characterization of virulence factors in *Escherichia coli* strains from patients with cystitis. *Rev. Inst. Med. Trop. Sao Paulo.* **2008**, *5*, 255–260. https://doi.org/10.1590/s0036-46652008000500001.
5. Luna-Pineda, V.M.; Ochoa, S.A.; Cruz-Córdova, A.; Cázares-Domínguez, V.; Reyes-Grajeda, J.P.; Flores-Oropeza, M.A.; Arellano-Galindo, J.; Hernández-Castro, R.; Flores-Encarnación, M.; Ramírez-Vargas, A.; et al. Correction: Features of urinary *Escherichia coli* isolated from children with complicated and uncomplicated urinary tract infections in Mexico. *PLoS ONE* **2018**, *13*, e0208285. https://doi.org/10.1371/journal.pone.0208285.
6. Saldaña, Z.; Xicohtencatl-Cortes, J.; Avelino, F.; Phillips, A.D.; Kaper, J.B.; Puente, J.L.; Girón, J.A. Synergistic role of curli and cellulose in cell adherence and biofilm formation of attaching and effacing *Escherichia coli* and identification of Fis as a negative regulator of curli. *Environ. Microbiol*. **2009**, *4*, 992–1006. https://doi.org/10.1111/j.1462-2920.2008.01824.x.
7. Saldaña, Z.; De la Cruz, M.A.; Carrillo-Casas, E.M.; Durán, L.; Zhang, Y.; Hernández-Castro, R.; Puente, J.L.; Daaka, Y.; Girón, J.A. Production of the Escherichia coli common pilus by uropathogenic *E. coli* is associated with adherence to HeLa and HTB-4 cells and invasion of mouse bladder urothelium. *PLoS ONE*. **2014**, *7*, e101200. https://doi.org/10.1371/journal.pone.0101200.
8. Gündoğdu, A.; Long, Y.B.; Vollmerhausen, T.L.; Katouli, M. Antimicrobial resistance and distribution of *sul* genes and integron-associated *intI* genes among uropathogenic *Escherichia coli* in Queensland, Australia. *J. Med. Microbiol.* **2011**, *60*, 1633–1642. https://doi.org/10.1099/jmm.0.034140-0.
9. Dillon, B.; Thomas, L.; Mohmand, G.; Zelynski, A.; Iredell, J. Multiplex PCR for screening of integrons in bacterial lysates. *J. Microbiol. Methods.* **2005**, *2*, 221–232. https://doi.org/10.1016/j.mimet.2005.02.007.
